# Ultrasound-guided hydrostatic reduction versus fluoroscopy-guided air reduction for pediatric intussusception: a multi-center, prospective, cohort study

**DOI:** 10.1186/s13017-020-00346-9

**Published:** 2021-01-13

**Authors:** Shu Ting Liu, Xiao Bing Tang, Huan Li, Dong Chen, Jun Lei, Yu Zuo Bai

**Affiliations:** 1 Department of Pediatric Surgery, Shengjing Hospital, China Medical University, No. 36 Sanhao Street, Heping District, Shenyang, 110004 P. R. China; 2 Department of Pediatric Surgery, Wuhan Children’s Hospital, Wuhan, China; 3 Department of Pediatric Surgery, Xi’an Children’s Hospital, Xi’an, China; 4 Department of Pediatric Surgery, Jiangxi Provincial Children’s Hospital, Nanchang, China; 5 The Pediatric Anorectal Group, Society of Pediatric Surgery, Chinese Medical Association, Shenyang, China

**Keywords:** Intussusception, Enema reduction, Multicenter study, Pediatric, Hydrostatic, Fluoroscopy

## Abstract

**Background:**

Intussusception is the most common abdominal emergency in children. The first line treatment of uncomplicated pediatric intussusception is enema reduction. Until now, there have been no multi-center studies comparing the effectiveness and safety of UGHR and FGAR in the treatment of pediatric intussusception. The aim of this study was to compare the effectiveness and safety of the two most commonly used enema methods of pediatric intussusception: ultrasound-guided hydrostatic reduction (UGHR) and fluoroscopy-guided air reduction (FGAR).

**Methods:**

From November 1, 2017 to October 31, 2018, we conducted a multi-center, prospective, cohort study. Children diagnosed with intussusception in four large Children’s Medical Centers in China were divided into UGHR and FGAR groups. Stratified analysis and subgroup analysis were used for further comparison. The success and recurrence rates were used to evaluate the effectiveness of enema reduction. The perforation rate was used to evaluate the safety of enema reduction.

**Results:**

A total of 2124 cases met the inclusion criteria (UGHR group: 1119 cases; FGAR group: 1005 cases). The success and recurrence rates in the UGHR group were higher than in the FGAR group (95.80%, 9.28% vs. 93.13%, 10.65%) (*P* < 0.05, *P* > 0.05), respectively. The perforation rate in the UGHR group was 0.36% compared with 0.30% in the FGAR group (*P* > 0.05). Subgroup analysis showed the success rates in the UGHR group were higher than in the FGAR group of patients with onset time between 12 and 24 h (95.56% vs. 90.57%) (*P* < 0.05). Of patients aged 4 to 24 months, the success rates in the UGHR group were also higher than in the FGAR group (95.77% vs. 91.60%) (*P* < 0.05). Stratified analysis showed the success rates in the UGHR group were higher than in the FGAR group in patients with the symptom of bloody stool (91.91% vs 85.38%) (*P* < 0.05).

**Conclusions:**

UGHR and FGAR are safe, nonsurgical treatment methods for acute pediatric intussusception. UGHR is superior to FGAR, no radiation risk, its success rate is higher, without a difference in perforation rate, especially for patients aged 4–24 months.

**Level of evidence:**

Level II.

## Introduction

Intussusception is one of the most common abdominal emergencies in infants and toddlers, typically occurring in infants between 4 and 10 months. After 2 years of age, the incidence of intussusception declines [1, 2]. Delayed diagnosis and treatment may lead to intestinal necrosis or even death. The definition of intussusception is given as the invagination of one segment of intestine into a segment of distal intestine. Common symptoms and signs include colicky abdominal pain, vomiting, palpable abdominal mass, and currant jelly stool.

The treatment methods of pediatric intussusception are divided into two types: surgical treatment or nonsurgical treatment. For uncomplicated pediatric intussusception, imaging-guided enema reduction is the internationally recognized, standard, nonsurgical treatment method [3], which can cure the vast majority of intussusception cases. Operation is mainly performed in patients for whom nonsurgical treatment has failed or those with complicated intussusception.

Both hydrostatic and air enemas can be used to reduce intussuscepted bowel, under the guidance of either fluoroscopy or ultrasonography. The preferred method of enema reduction is not standardized. There is practice variability among different institutions regarding to the type of enema (air or liquid) used and different international guidelines. Currently, ultrasound-guided hydrostatic reduction (UGHR) and fluoroscopy-guided air reduction (FGAR) are the most commonly used nonsurgical treatment methods [4, 5], the indications and contraindications of which are basically the same. There are only a few studies comparing the effectiveness and safety of UGHR and FGAR in the literature [6–9], and no clear consensus has been reached regarding the optimal reduction strategy. Some studies demonstrated a higher success rate of UGHR [7, 8], while some studies demonstrated a higher success rate of FGAR [6, 9]. Till now, there was no multi-center prospective study for UGHR and FGAR in the treatment of pediatric intussusception in the world.

In order to find the optimal treatment method for pediatric intussusception, we conducted a multi-center, prospective clinical study with a large number of intussusception cases to compare the effectiveness and safety of UGHR and FGAR in the treatment of pediatric intussusception.

## Methods

### Study design

This was a multi-center, prospective, observational cohort study with the purpose of comparing the effectiveness and safety of UGHR and FGAR in pediatric intussusception patients. It included four large children’s medical centers from different regions of China: Shengjing Hospital of China Medical University, Xi’an Children’s Hospital, Wuhan Children’s Hospital, and Jiangxi Provincial Children’s Hospital. This study was approved by the Institutional Review Board of Shengjing Hospital of China Medical University (Approval No.2019PS601K), and has been exempted from the application for informed consent.

### Participants and data collection

Patients under 14 years old who were diagnosed with intussusception (ICD-10 code K56.1) and underwent enema reduction between November 1, 2017 and October 31, 2018 were entered into this study.

The data inclusion criteria were as follows: (1) intussusception is diagnosed by ultrasound with characteristic image; (2) the onset time is less than 48 h; (3) aged between 4 months and 14 years; (4) a well general condition and no signs of peritonitis; (5) no clinical manifestations of small intestinal obstruction; and (6) patients who suffer from intussusception again within 1 month are considered as recurrence of intussusception.

Patients’ clinical data was recorded, including patient’s gender, age, admission time, onset time, main symptoms, concentric circle size on ultrasound, and outcome of enema reduction.

### Grouping

According to the conventional enema reduction method used in the four hospitals, the cases were divided into two groups: a UGHR group and a FGAR group. Shengjing Hospital of China Medical University and Jiangxi Provincial Children’s Hospital applied UGHR; Xi’an Children’s Hospital and Wuhan Children’s Hospital applied FGAR. UGHR or FGAR is the conventional treatment methods in these hospitals, and implementation of this study does not require any special changes to the treatment methods.

In order to minimize the impact of age and time of onset on the results of this study, we conducted a stratified analysis of patient data. The data was divided into subgroups according to the onset time and age. Stratified analysis was made according to the symptom of blood stool.

### Enema reduction procedures

The procedure of UGHR [10]: reduction was performed by two pediatric surgeons using ultrasound guidance. Patients were placed in a supine position, a Foley catheter was inserted via the anus, and the buttock was taped to prevent normal saline leakage. Under ultrasonography guidance, normal saline solution (37 °C to 40 °C) was manually injected through the Foley catheter. The hydrostatic pressure was monitored by a sphygmomanometer attached to the Foley catheter. The maximum pressure was controlled under 100 mmHg. The success of reduction was determined by the disappearance of intussusception and the visualization of normal saline from the cecum to the ileum through the ileocecal valve or a normal saline distended ileum.

The procedure of FGAR: reduction was performed by a radiologist in the company of a pediatric surgeon using fluoroscopic guidance. Patients were placed in a supine position, a Foley catheter was inserted via the anus, and the buttock was taped to prevent air leakage. Under the fluoroscopic monitoring, air was injected through the Foley catheter. Pressure between 80 and 100 mmHg, controlled by a barometer, was applied. The success of reduction was determined by the disappearance of intussusception and the visualization of air from the cecum to the ileum through the ileocecal valve or an air-distended ileum.

Both of these two procedures were repeated no more than 3 attempts.

### Post-procedure treatments

All patients in both treatment groups underwent repeated ultrasound to confirm the success of the enema reduction. After a successful reduction, the patient needs to be hospitalized for about 12 h until the next normal defecation.

### Effectiveness and safety assessment

The success and recurrence rates were used to evaluate the effectiveness of enema reduction. The perforation rate was used to evaluate the safety of enema reduction. The efficacy of different treatment groups are further compared through subgroup and stratified analysis.

### Statistical analysis

Data analysis was done using the data analysis function of Microsoft Office Excel (version OFFICE 2019 DESKTOP @Microsoft Corporation). Numerical descriptive data were presented as mean and standard deviation. The categorical descriptive data were reported as numbers (*N*) and percentages (%). Comparisons between the two groups were made using the Chi-square test and the Fisher test for T (theoretical frequency) < 5 or *n* (total number) < 40. A value of *P* < 0.05 was considered statistically significant.

## Results

**Fig. 1:**
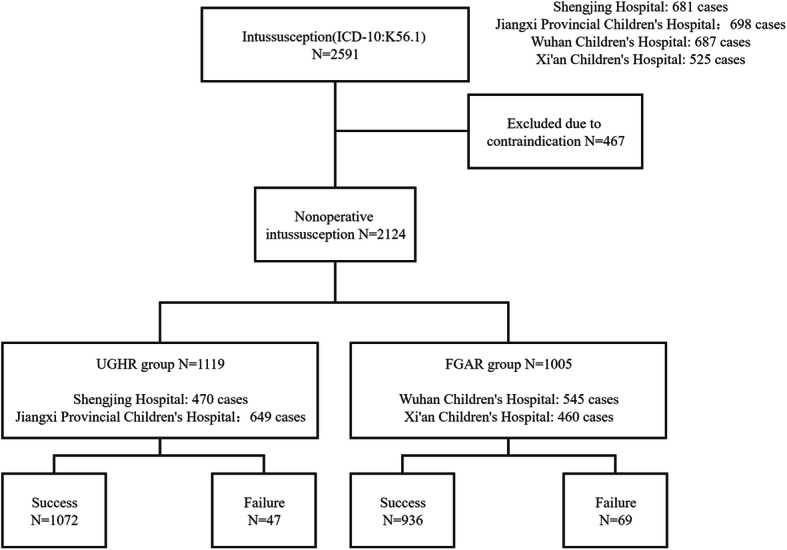
Study flowchart

From November 2017 to October 2018, a total of 2591 intussusception cases were collected, according to the inclusion criteria; 2124 cases were finally enrolled in this study, including 1119 cases in the UGHR group and 1005 cases in the FGAR group (Fig. 1). The male to female ratio was 2:1. The patients’ median age was 23.66 months. There was no significant difference in age (*P* = 0.28) or gender (*P* = 0.52) between the two groups (Table 1).

**Table 1:** Comparison between the UGHR and FGAR group

	UGHR	FGAR	Total	*P* value
Total cases, *n*	1119	1005	2124	
Male, *n* (%)	731 (65.33%)	670 (66.67%)	1401	0.5151
Female, *n* (%)	388 (34.67%)	335 (33.33%)	723	
Mean age, month (range)	23.2 (4–156)	24.7 (4–146)	23.66	0.2770
Mean onset time, month	17.2	19.6	18.36	
Success cases, *n* (%)	1072 (95.80%)	936 (93.13%)	2008 (94.54%)	0.0069
Recurrence cases, *n* (%)	104 (9.28%)	107 (10.65%)	211 (9.93%)	0.2980
Perforation cases, *n* (%)	4 (0.36%)	3 (0.30%)	7 (0.33%)	0.9325

The overall success rate in this study was 94.54%. The success rate of the UGHR group (95.80%) was higher than that of the FGAR group (93.13%) (*P* = 0.007). The overall recurrence rate in this study was 9.93%. The recurrence rate in the UGHR group (9.28%) was lower than that of the FGAR group (10.65%), but it was not statistically significant (*P* = 0.2980) (Table 1).

The overall perforation rate in this study was 0.33%. The perforation rate in the UGHR group was 0.36% higher than the FGAR group at 0.30%, but the difference was not statistically significant (*P* = 0.9325) (Table 1).

In order to analyze the effectiveness of UGHR and FGAR in patients with different onset times, the cases were divided into three subgroups according to the onset time: less than 12 h, 12 h to 24 h, and more than 24 h. The success rate of UGHR group (95.56%) was higher than that of the FGAR group (90.57%) in the 12 h to 24 h group (*P* = 0.0049). There was no significant difference in the groups with onset times less than 12 h and more than 24 h (Table 2).

**Table 2:** Success rate in subgroups according to onset time

Group	Outcome	UGHR	FGAR	Total	*P* value
0–12 h	Success	504 (97.30%)	392 (96.80)	896	0.6500
	Fail	14	13	27	
12h–24 h	Success	387 (95.56%)	384 (90.57%)	771	0.0049
	Fail	18	40	58	
> 24 h	Success	181 (92.35%)	160 (90.91%)	341	0.6164
	Fail	15	16	31	

In order to analyze the effectiveness of UGHR and FGAR in patients of different ages, the cases were divided into two groups according to age: 4 to 24 months, and older than 24 months. The success rate of UGHR group was higher than that of FGAR group in age group 4 to 24 months (*P* = 0.0013). The group older than 24 months showed no significant differences in the success rate (Table 3).

**Table 3:** Success rate in subgroups according to age

Group	Outcome	UGHR	FGAR	Total	*P* value
4–24 months	Success	702 (95.77%)	600(91.60%)	1302	0.0013
	Fail	31	55	86	
> 24 months	Success	370 (95.85%)	336(96.00%)	706	0.9208
	Fail	16	14	30	

We further analyzed patients with the symptom of bloody stool as a stratified analysis. The success rate of the UGHR group (91.91%) was higher than that of FGAR group (85.38%) (*P* = 0.0085) (Table 4). The recurrence rate of the UGHR group (3.46%) was lower than that of the FGAR group (6.61%), but it did not have significant statistical difference (*P* = 0.0804).

**Table 4:** Success rate and recurrence rate in patients with bloody stool

	UGHR	FGAR	Total	*P* value
Success cases, *n* (%)	318 (91.91%)	257 (85.38%)	575 (88.87%)	0.0085
Recurrence cases, *n* (%)	11/318 (3.46%)	17/257 (6.61%)	28/575 (4.87%)	0.0804
Total cases, *n* (%)	346	301	649	

## Discussion

UGHR and FGAR are two most commonly used nonsurgical treatment methods of uncomplicated pediatric intussusception [4, 5, 11]. However, which of these two methods is more suitable for intussusception remains controversial. This research is the first multi-center and prospective study of enema reduction for the treatment of pediatric intussusception in the world. The results of this study showed that the success rate of UGHR is higher than FGAR, without significant difference in recurrence rate and perforation rate (Table 1). This demonstrates that UGHR is more effective than FGAR; and UGHR and FGAR are both safe methods for treatment of pediatric intussusception.

Intussusception is more common in children under 2 years of age, which may be caused by various etiology [12]. After 2 years of age, the incidence of intussusception declines. Therefore, stratified study was performed according to age and demonstrates that UGHR is more effective than FGAR for intussusception cases aged 4 to 24 months (Table 3). So, we considered that UGHR is more suitable than FGAR for intussusception patients under 2 years old.

In patients with intussusception, the edema and ischemia of the digestive tract worse with time, and the risk of perforation caused by enema treatment increases with time [1]. Therefore, we conducted a stratified analysis according to onset time and demonstrates that UGHR is more effective than FGAR for intussusception cases in the 12 h to 24 h group, while has no inferior efficacy to FGAR in other groups (Table 2). So, we considered that UGHR is more suitable than FGAR for intussusception patients with onset time between 12 and 24 h.

Previous studies have shown that bloody stool is one of the risk factors associated with recurrence of pediatric intussusception [13, 14], and also indicates the severity of intussusception [1], which means that children with bloody stools have a higher risk of nonsurgical treatment failure. Therefore, we conducted a subgroup analysis of intussusception cases with the symptom of bloody stools and demonstrate that UGHR is more effective than FGAR (Table 4). So, we considered that UGHR is more suitable than FGAR for intussusception patients with the symptom of bloody stools.

Until now, the studies including a large number of cases in the treatment of pediatric intussusception were almost retrospective single-center study with only one enema reduction method [10, 15, 16]. There were a few original studies comparing the effectiveness of UGHR and FGAR in the treatment of pediatric intussusception [4, 6]. A randomized controlled trial (RCT) in 2018 of 124 pediatric intussusception cases in China showed that the success rate of UGHR (96.77%) was higher than FGAR (83.87%), which demonstrated that UGHR is more effective than FGAR [8]. A prospective cohort study in 2017 of 80 pediatric intussusception cases in Egypt showed that the success rates of FGAR and UGHR were equal (82.5%), which demonstrated a similar effectiveness of UGHR and FGAR [6]. Both of these two studies had the disadvantage of a small number of cases (less than 200 cases). In this study, we collected more than 2000 cases of pediatric intussusception to compare the effectiveness and safety of UGHR and FGAR. The four Children’s Medical Centers participating in this study are distributed in the northeast, southeast, western, and central regions of China, and the geographical distribution is relatively average. Therefore, this study avoids differences in region, culture, and lifestyle; thereby making the final results more credible and representative of the characteristics of Chinese pediatric intussusception cases.

FGAR has gained widespread acceptance worldwide as it has several advantages: easy to perform, quick, and clean [17].

Compared with FGAR, UGHR has some advantages. First, ultrasound can clearly show intussusception masses (including edema of ileocecal valve) and can detect pathologically induced point or residual intussusception early [18, 19]. Previous study have showed ultrasound examination has significant advantages over fluoroscopy in terms of diagnostic specificity and sensitivity of intussusception [20]. This ensures patients receive accurate treatment as early as possible.

Second, UGHR is completely free of ionizing radiation, which is the main disadvantage of FGAR. Early studies focused less on radiation dose during enema reduction under fluoroscopy. Some studies show that the radiation dose of enema reduction under fluoroscopy one time is not enough to cause significant harm to the human body [21, 22]. The small effect of ionizing radiation on the human body still has unexpected hazards, especially in children whose glands are more sensitive to radiation [23]. Acute pediatric intussusception is a common abdominal condition with a high rate of recurrence. In this study, the overall recurrence rate is 9.93% (Table 1). Therefore, this procedure often requires repeating. Under FGAR, the intussusception patients, their parents, and the medical staff can be exposed to ionizing radiation multiple times. The accumulation of radiation in the human body within a short period of time may also cause pathological changes; exact research has shown that receiving large doses of electromagnetic radiation can cause radiation-related malignancy [24, 25]. UGHR totally avoids radiation damage to human health, which is very meaningful for the protection of the patients, parents of the patients, and medical staff.

Despite the above-mentioned advantages, UGHR is not very widely applied because it requires special training. Our status survey in 2019 on enema reduction of pediatric intussusception in China showed that only 17.2% (22/128) hospitals used ultrasound to monitor the enema reduction, and pediatric surgeons were solely responsible for performing UGHR in only 36.4% (8/22) of these hospitals [4]. In Germany, pediatric surgeons can routinely use ultrasound to diagnose typical pediatric surgical diseases (including appendicitis and intussusception), and solely operate UGHR without the presence of an ultrasonographer [26]. In China, UGHR is not a routine training for pediatric residents, so most pediatric surgeons cannot perform UGHR alone. Having pediatric surgeons present to immediately judge and deal with unexpected intestinal perforation during enema can maximally decrease the delay of surgical treatment. Moreover, studies in the United Kingdom and Japan have supported the active role of the pediatric surgeon during enema reduction [7, 27]. We believe that Chinese pediatric surgery residents also need ultrasound training, not only for treating intussusception, but also in clinical practice of other common disease.

## Limitation

Some limitations of the present study should be acknowledged. First, this study is a prospective cohort study rather than a randomized control trial (RCT) comparing UGHR and FGAR. This may lead to potential selectivity bias and has a lower quality of evidence than RCT, but this error is much less than in a single-center study. Second, the time range of this study is relatively short (between November 2017 and October 2018) and the number of cases included in the study was less than expected; extending the research time and increasing the number of cases may increase the quality of the research evidence. Thirdly, as a multi-center study, the data of the four participating medical centers may have technical bias, but the participating institutions are all at the first-line level of pediatric intussusception treatment in China. The mature technology and trained doctors can minimize such bias.

## Conclusions

In conclusion, UGHR and FGAR are both safe, nonsurgical treatment methods for acute pediatric intussusception. UGHR is superior to FGAR, no radiation risk, and the success rate is higher without a difference in perforation rate, especially for patients aged 4 to 24 months.

## Data Availability

All data generated or analyzed during this study are included in this published article.

## Abbreviations

UGHR: Ultrasound-guided hydrostatic reduction
FGAR: Fluoroscopy-guided air reduction
